# Supplementary material for: Conservation of carbon resources and values on public lands: A case study from the National Wildlife Refuge System
Source: PLoS One. 2022 Jan 12;17(1):e0262218. doi: 10.1371/journal.pone.0262218 (PMC8754292; doi:10.1371/journal.pone.0262218)
Supplement: S1 Table — Valuation for social cost of carbon based on the Interagency Working Group on social cost of carbon values [30] escalated to 2018 USD using the Bureau of Labor Statistics Consumer Price Index and the net ecosystem carbon balance. Values represent net present value of damages avoided at the 5, 3, and 2.5 percent discount rates for average climate change scenario damages and at the 3% discount rate for the 95th percentile climate change scenario damages. (DOCX) [file pone.0262218.s001.docx]

**S1 Table**. **Mean carbon stock and mean net ecosystem carbon balance in and outside of the national wildlife refuges studied, total carbon dioxide sequestered, and associated social cost of carbon for National Wildlife Refuges. Valuation for social cost of carbon based on the Interagency Working Group on social cost of carbon values [30] escalated to 2018 USD using the Bureau of Labor Statistics Consumer Price Index and the net ecosystem carbon balance. Values represent net present value of damages avoided at the 5, 3, and 2.5 percent discount rates for average climate change scenario damages and at the 3% discount rate for the 95^th^ percentile climate change scenario damages.**

| National Wildlife Refuge Name | Mean carbon Stock in NWR (gCm^-2^) | Mean NECB in NWR (gCm^-2^yr^-1^) | Mean carbon stock outside NWR (gCm^-2^) | Mean NECB outside NWR (gCm^-2^yr^-1^) | Carbon Dioxide sequestered ton CO2/yr | Social Cost of Carbon  (Millions of 2018 USD/year) | | | |
| --- | --- | --- | --- | --- | --- | --- | --- | --- | --- |
|  |  |  |  |  |  | 5% discount rate at average damages scenario | 3% discount rate at average damages scenario | 2.5% discount rate at average damages scenario | 3% discount rate at 95th percentile damages scenario |
| Alaska Maritime National Wildlife Refuge | 113,907 | 49 | 31 | 84,043 | 3,288,234 | 47,745,152 | 159,150,506 | 238,725,759 | 461,536,468 |
| Alaska Peninsula National Wildlife Refuge | 115,376 | 53 | 49 | 116,041 | 3,076,674 | 44,673,302 | 148,911,007 | 223,366,510 | 431,841,919 |
| Okefenokee National Wildlife Refuge | 24,754 | 294 | 310 | 17,565 | 1,781,909 | 25,873,324 | 86,244,413 | 129,366,619 | 250,108,797 |
| Kenai National Wildlife Refuge | 71,871 | 43 | 32 | 68,990 | 1,247,126 | 18,108,266 | 60,360,885 | 90,541,328 | 175,046,567 |
| Togiak National Wildlife Refuge | 71,229 | 16 | 10 | 57,771 | 1,026,266 | 14,901,387 | 49,671,291 | 74,506,937 | 144,046,745 |
| Yukon Delta National Wildlife Refuge | 46,901 | 3 | 2 | 46,577 | 979,666 | 14,224,748 | 47,415,827 | 71,123,740 | 137,505,898 |
| Becharof National Wildlife Refuge | 95,416 | 37 | 23 | 84,827 | 798,516 | 11,594,452 | 38,648,174 | 57,972,260 | 112,079,703 |
| Kodiak National Wildlife Refuge | 87,364 | 31 | 21 | 77,970 | 760,018 | 11,035,461 | 36,784,871 | 55,177,307 | 106,676,127 |
| Alligator River National Wildlife Refuge | 32,738 | 316 | 219 | 30,338 | 721,293 | 10,473,175 | 34,910,583 | 52,365,875 | 101,240,692 |
| Desert National Wildlife Range | 1,424 | 26 | 23 | 1,371 | 632,979 | 9,190,861 | 30,636,205 | 45,954,307 | 88,844,994 |
| Arctic National Wildlife Refuge | 40,362 | 2 | (4) | 38,430 | 500,079 | 7,261,149 | 24,203,829 | 36,305,744 | 70,191,105 |
| Kofa National Wildlife Refuge | 1,238 | 45 | 24 | 1,103 | 448,492 | 6,512,107 | 21,707,025 | 32,560,537 | 62,950,371 |
| Pocosin Lakes National Wildlife Refuge | 31,820 | 249 | 216 | 26,385 | 422,485 | 6,134,482 | 20,448,273 | 30,672,409 | 59,299,990 |
| Dale Bumpers White River National Wildlife Refuge | 11,460 | 170 | 130 | 8,023 | 398,581 | 5,787,392 | 19,291,308 | 28,936,962 | 55,944,793 |
| St. Marks National Wildlife Refuge | 15,360 | 329 | 350 | 16,044 | 371,591 | 5,395,506 | 17,985,018 | 26,977,528 | 52,156,553 |
| Merritt Island National Wildlife Refuge | 8,497 | 154 | 104 | 8,588 | 295,367 | 4,288,726 | 14,295,755 | 21,443,632 | 41,457,689 |
| Aransas National Wildlife Refuge | 3,555 | 164 | 136 | 4,060 | 284,055 | 4,124,486 | 13,748,286 | 20,622,429 | 39,870,030 |
| Lower Suwannee National Wildlife Refuge | 21,263 | 311 | 276 | 15,279 | 239,798 | 3,481,863 | 11,606,209 | 17,409,313 | 33,658,005 |
| Carolina Sandhills National Wildlife Refuge | 14,872 | 347 | 243 | 9,755 | 235,915 | 3,425,483 | 11,418,277 | 17,127,415 | 33,113,003 |
| Izembek National Wildlife Refuge | 109,889 | 38 | 42 | 117,353 | 231,401 | 3,359,945 | 11,199,818 | 16,799,727 | 32,479,471 |
| Great Dismal Swamp National Wildlife Refuge | 32,666 | 136 | 79 | 17,058 | 228,037 | 3,311,091 | 11,036,969 | 16,555,454 | 32,007,211 |
| Stillwater National Wildlife Refuge | 3,599 | 179 | 28 | 2,057 | 219,240 | 3,183,368 | 10,611,228 | 15,916,842 | 30,772,561 |
| Cache River National Wildlife Refuge | 6,636 | 180 | 160 | 6,414 | 191,294 | 2,777,582 | 9,258,606 | 13,887,909 | 26,849,957 |
| Felsenthal National Wildlife Refuge | 11,866 | 189 | 156 | 9,844 | 187,919 | 2,728,582 | 9,095,273 | 13,642,910 | 26,376,292 |
| Seney National Wildlife Refuge | 18,563 | 129 | 212 | 17,418 | 182,924 | 2,656,063 | 8,853,545 | 13,280,317 | 25,675,279 |
| Ten Thousand Islands National Wildlife Refuge | 24,537 | 354 | 234 | 14,104 | 178,334 | 2,589,410 | 8,631,368 | 12,947,052 | 25,030,967 |
| Tensas River National Wildlife Refuge | 9,631 | 152 | 115 | 7,417 | 176,454 | 2,562,112 | 8,540,372 | 12,810,558 | 24,767,079 |
| Tennessee National Wildlife Refuge | 11,407 | 243 | 259 | 9,136 | 173,333 | 2,516,798 | 8,389,327 | 12,583,990 | 24,329,048 |
| St. Vincent National Wildlife Refuge | 13,551 | 903 | 571 | 20,492 | 163,290 | 2,370,977 | 7,903,256 | 11,854,884 | 22,919,442 |
| Sam D. Hamilton Noxubee National Wildlife Refuge | 13,421 | 227 | 174 | 10,304 | 161,977 | 2,351,904 | 7,839,681 | 11,759,522 | 22,735,075 |
| Waccamaw National Wildlife Refuge | 19,674 | 312 | 255 | 15,206 | 159,452 | 2,315,237 | 7,717,455 | 11,576,183 | 22,380,621 |
| Upper Ouachita National Wildlife Refuge | 9,847 | 196 | 171 | 9,895 | 158,168 | 2,296,598 | 7,655,327 | 11,482,991 | 22,200,449 |
| Cape Romain National Wildlife Refuge | 5,359 | 168 | 137 | 12,013 | 152,877 | 2,219,770 | 7,399,235 | 11,098,852 | 21,457,781 |
| Bogue Chitto National Wildlife Refuge | 11,660 | 274 | 267 | 10,658 | 145,258 | 2,109,150 | 7,030,499 | 10,545,749 | 20,388,447 |
| Piedmont National Wildlife Refuge | 13,508 | 273 | 277 | 11,297 | 141,390 | 2,052,989 | 6,843,296 | 10,264,945 | 19,845,560 |
| Mingo National Wildlife Refuge | 10,703 | 398 | 120 | 8,330 | 127,998 | 1,858,524 | 6,195,080 | 9,292,621 | 17,965,733 |
| Upper Mississippi River National Wildlife And Fish Refuge | 9,748 | 45 | 36 | 6,462 | 125,256 | 1,818,722 | 6,062,406 | 9,093,610 | 17,580,979 |
| San Bernard National Wildlife Refuge | 10,767 | 136 | 125 | 8,710 | 124,460 | 1,807,158 | 6,023,860 | 9,035,791 | 17,469,195 |
| Chickasaw National Wildlife Refuge | 11,453 | 309 | 176 | 7,154 | 121,107 | 1,758,477 | 5,861,591 | 8,792,387 | 16,998,615 |
| Little Pend Oreille National Wildlife Refuge | 12,929 | 199 | 164 | 10,791 | 120,689 | 1,752,401 | 5,841,337 | 8,762,006 | 16,939,878 |
| Klamath Marsh National Wildlife Refuge | 10,042 | 194 | 114 | 8,169 | 119,276 | 1,731,892 | 5,772,974 | 8,659,462 | 16,741,626 |
| Nowitna National Wildlife Refuge | 20,602 | 4 | 8 | 20,573 | 113,641 | 1,650,070 | 5,500,234 | 8,250,350 | 15,950,677 |
| Crab Orchard National Wildlife Refuge | 7,916 | 163 | 113 | 6,747 | 109,661 | 1,592,275 | 5,307,583 | 7,961,375 | 15,391,992 |
| Trinity River National Wildlife Refuge | 9,830 | 249 | 200 | 8,677 | 107,467 | 1,560,423 | 5,201,410 | 7,802,115 | 15,084,090 |
| Hart Mountain National Antelope Refuge | 3,326 | 26 | 22 | 2,713 | 105,466 | 1,531,361 | 5,104,537 | 7,656,805 | 14,803,156 |
| Silvio O. Conte National Fish And Wildlife Refuge | 13,894 | 187 | 203 | 14,251 | 105,032 | 1,525,058 | 5,083,525 | 7,625,288 | 14,742,223 |
| Delta National Wildlife Refuge | 8,743 | 135 | 87 | 8,805 | 100,371 | 1,457,391 | 4,857,970 | 7,286,955 | 14,088,113 |
| Wheeler National Wildlife Refuge | 11,359 | 199 | 170 | 9,466 | 100,189 | 1,454,749 | 4,849,164 | 7,273,746 | 14,062,575 |
| Savannah National Wildlife Refuge | 17,321 | 203 | 188 | 13,481 | 95,426 | 1,385,587 | 4,618,623 | 6,927,935 | 13,394,007 |
| Balcones Canyonlands National Wildlife Refuge | 6,092 | 249 | 217 | 5,420 | 93,909 | 1,363,565 | 4,545,216 | 6,817,824 | 13,181,125 |
| Laguna Atascosa National Wildlife Refuge | 3,046 | 64 | 79 | 3,483 | 90,577 | 1,315,177 | 4,383,924 | 6,575,887 | 12,713,381 |
| Big Branch Marsh National Wildlife Refuge | 13,584 | 332 | 170 | 13,370 | 89,316 | 1,296,867 | 4,322,891 | 6,484,336 | 12,536,383 |
| Panther Swamp National Wildlife Refuge | 10,604 | 141 | 104 | 7,694 | 85,651 | 1,243,659 | 4,145,529 | 6,218,294 | 12,022,034 |
| Willapa National Wildlife Refuge | 18,047 | 344 | 159 | 23,715 | 84,795 | 1,231,220 | 4,104,067 | 6,156,101 | 11,901,796 |
| Florida Panther National Wildlife Refuge | 9,964 | 205 | 139 | 7,351 | 80,608 | 1,170,426 | 3,901,421 | 5,852,132 | 11,314,121 |
| Moosehorn National Wildlife Refuge | 14,431 | 180 | 190 | 14,307 | 79,200 | 1,149,991 | 3,833,304 | 5,749,955 | 11,116,580 |
| Edwin B. Forsythe National Wildlife Refuge | 20,817 | 120 | 125 | 17,351 | 78,160 | 1,134,887 | 3,782,956 | 5,674,435 | 10,970,573 |
| Hatchie National Wildlife Refuge | 11,749 | 446 | 265 | 8,505 | 75,668 | 1,098,703 | 3,662,344 | 5,493,516 | 10,620,798 |
| Lewis And Clark National Wildlife Refuge | 13,163 | 381 | 53 | 18,419 | 74,405 | 1,080,365 | 3,601,216 | 5,401,823 | 10,443,525 |
| Umbagog National Wildlife Refuge | 20,800 | 131 | 111 | 17,533 | 72,498 | 1,052,674 | 3,508,912 | 5,263,368 | 10,175,846 |
| Brazoria National Wildlife Refuge | 6,369 | 102 | 95 | 6,722 | 71,271 | 1,034,861 | 3,449,537 | 5,174,306 | 10,003,658 |
| Sacramento National Wildlife Refuge | 8,067 | 430 | 361 | 8,013 | 69,627 | 1,010,984 | 3,369,945 | 5,054,918 | 9,772,841 |
| Wichita Mountains Wildlife Refuge | 2,636 | 78 | 54 | 3,084 | 68,163 | 989,726 | 3,299,086 | 4,948,629 | 9,567,349 |
| Sevilleta National Wildlife Refuge | 1,833 | 20 | 22 | 2,074 | 67,632 | 982,021 | 3,273,404 | 4,910,106 | 9,492,871 |
| Lower Klamath National Wildlife Refuge | 5,538 | 88 | 29 | 5,215 | 67,440 | 979,234 | 3,264,113 | 4,896,170 | 9,465,928 |
| Lower Rio Grande Valley National Wildlife Refuge | 3,395 | 46 | 18 | 3,204 | 65,627 | 952,907 | 3,176,357 | 4,764,536 | 9,211,436 |
| Tishomingo National Wildlife Refuge | 5,172 | 266 | 215 | 4,514 | 64,999 | 943,786 | 3,145,954 | 4,718,931 | 9,123,266 |
| Mississippi Sandhill Crane National Wildlife Refuge | 13,001 | 217 | 355 | 15,266 | 64,952 | 943,102 | 3,143,672 | 4,715,508 | 9,116,648 |
| Saddle Mountain National Wildlife Refuge | 2,950 | 27 | 27 | 2,824 | 64,812 | 941,066 | 3,136,886 | 4,705,329 | 9,096,970 |
| Anahuac National Wildlife Refuge | 9,459 | 112 | 91 | 7,752 | 64,020 | 929,565 | 3,098,551 | 4,647,826 | 8,985,796 |
| Sacramento River National Wildlife Refuge | 8,639 | 367 | 383 | 8,177 | 63,451 | 921,305 | 3,071,017 | 4,606,525 | 8,905,949 |
| Delevan National Wildlife Refuge | 12,046 | 723 | 418 | 7,846 | 62,203 | 903,185 | 3,010,617 | 4,515,925 | 8,730,789 |
| Big Lake National Wildlife Refuge | 11,121 | 380 | 260 | 8,069 | 61,417 | 891,776 | 2,972,586 | 4,458,879 | 8,620,499 |
| Pond Creek National Wildlife Refuge | 10,239 | 145 | 89 | 6,952 | 61,344 | 890,716 | 2,969,052 | 4,453,579 | 8,610,252 |
| San Pablo Bay National Wildlife Refuge | 11,795 | 203 | 121 | 6,720 | 60,534 | 878,956 | 2,929,853 | 4,394,780 | 8,496,575 |
| Lower Hatchie National Wildlife Refuge | 9,673 | 286 | 246 | 9,143 | 59,971 | 870,784 | 2,902,613 | 4,353,919 | 8,417,577 |
| Sonny Bono Salton Sea National Wildlife Refuge | 3,181 | 107 | 101 | 2,455 | 58,390 | 847,826 | 2,826,087 | 4,239,130 | 8,195,652 |
| Atchafalaya National Wildlife Refuge | 11,944 | 248 | 197 | 10,340 | 57,981 | 841,877 | 2,806,256 | 4,209,384 | 8,138,143 |
| Catahoula National Wildlife Refuge | 6,554 | 152 | 177 | 9,443 | 56,949 | 826,905 | 2,756,351 | 4,134,526 | 7,993,417 |
| D 'Arbonne National Wildlife Refuge | 10,730 | 217 | 178 | 10,257 | 56,915 | 826,404 | 2,754,681 | 4,132,022 | 7,988,575 |
| Sheldon National Wildlife Refuge | 2,657 | 6 | 18 | 2,677 | 54,773 | 795,299 | 2,650,998 | 3,976,497 | 7,687,894 |
| Roanoke River National Wildlife Refuge | 19,492 | 158 | 151 | 12,694 | 50,006 | 726,082 | 2,420,275 | 3,630,412 | 7,018,797 |
| Lake Woodruff National Wildlife Refuge | 17,095 | 151 | 238 | 17,270 | 48,283 | 701,069 | 2,336,895 | 3,505,343 | 6,776,996 |
| Santee National Wildlife Refuge | 10,955 | 233 | 120 | 9,392 | 44,698 | 649,010 | 2,163,365 | 3,245,048 | 6,273,759 |
| Mattamuskeet National Wildlife Refuge | 14,680 | 60 | 133 | 23,007 | 44,504 | 646,201 | 2,154,002 | 3,231,003 | 6,246,606 |
| Hagerman National Wildlife Refuge | 5,293 | 265 | 202 | 3,711 | 44,477 | 645,804 | 2,152,681 | 3,229,021 | 6,242,774 |
| Cross Creeks National Wildlife Refuge | 11,355 | 333 | 252 | 9,701 | 43,558 | 632,456 | 2,108,185 | 3,162,278 | 6,113,738 |
| Hillside National Wildlife Refuge | 10,610 | 188 | 182 | 9,610 | 43,221 | 627,568 | 2,091,892 | 3,137,838 | 6,066,487 |
| Clarks River National Wildlife Refuge | 8,852 | 300 | 175 | 6,683 | 41,660 | 604,910 | 2,016,368 | 3,024,552 | 5,847,467 |
| Lake Ophelia National Wildlife Refuge | 10,306 | 152 | 166 | 10,260 | 39,206 | 569,276 | 1,897,585 | 2,846,378 | 5,502,997 |
| Reelfoot National Wildlife Refuge | 11,334 | 250 | 222 | 8,731 | 38,709 | 562,051 | 1,873,503 | 2,810,254 | 5,433,157 |
| Upper Klamath National Wildlife Refuge | 18,752 | 110 | 132 | 14,362 | 37,905 | 550,381 | 1,834,604 | 2,751,907 | 5,320,353 |
| Blackwater National Wildlife Refuge | 20,891 | 85 | 120 | 12,047 | 37,766 | 548,359 | 1,827,862 | 2,741,794 | 5,300,801 |
| Blackbeard Island National Wildlife Refuge | 9,626 | 455 | 352 | 19,422 | 37,746 | 548,077 | 1,826,923 | 2,740,385 | 5,298,078 |
| Malheur National Wildlife Refuge | 4,103 | 13 | 38 | 3,874 | 37,096 | 538,637 | 1,795,455 | 2,693,183 | 5,206,820 |
| Chincoteague National Wildlife Refuge | 10,636 | 188 | 121 | 8,466 | 36,800 | 534,342 | 1,781,139 | 2,671,708 | 5,165,302 |
| Pee Dee National Wildlife Refuge | 14,312 | 286 | 270 | 10,741 | 36,681 | 532,612 | 1,775,372 | 2,663,058 | 5,148,579 |
| Sunkhaze Meadows National Wildlife Refuge | 17,445 | 209 | 213 | 16,864 | 36,133 | 524,647 | 1,748,825 | 2,623,237 | 5,071,591 |
| Mountain Longleaf National Wildlife Refuge | 11,924 | 267 | 201 | 9,356 | 35,693 | 518,266 | 1,727,554 | 2,591,330 | 5,009,905 |
| St. Catherine Creek National Wildlife Refuge | 7,631 | 93 | 170 | 9,862 | 35,096 | 509,587 | 1,698,625 | 2,547,937 | 4,926,011 |
| Little River National Wildlife Refuge | 8,892 | 162 | 120 | 6,152 | 34,296 | 497,980 | 1,659,934 | 2,489,902 | 4,813,810 |
| Crescent Lake National Wildlife Refuge | 2,785 | 50 | 40 | 2,367 | 34,256 | 497,403 | 1,658,010 | 2,487,015 | 4,808,230 |
| Bayou Sauvage National Wildlife Refuge | 10,500 | 89 | 30 | 10,386 | 33,639 | 488,444 | 1,628,148 | 2,442,222 | 4,721,628 |
| Swan Lake National Wildlife Refuge | 6,177 | 196 | 93 | 5,949 | 32,073 | 465,704 | 1,552,348 | 2,328,521 | 4,501,808 |
| San Andres National Wildlife Refuge | 2,061 | 37 | 36 | 1,802 | 31,063 | 451,039 | 1,503,463 | 2,255,194 | 4,360,042 |
| Julia Butler Hansen Refuge For The Columbian White-Tailed Deer | 11,135 | 344 | 33 | 9,179 | 30,899 | 448,652 | 1,495,507 | 2,243,260 | 4,336,970 |
| National Elk Refuge | 4,392 | 83 | 89 | 4,857 | 30,706 | 445,852 | 1,486,174 | 2,229,260 | 4,309,903 |
| Mcfaddin National Wildlife Refuge | 9,426 | 31 | 69 | 8,756 | 30,693 | 445,655 | 1,485,518 | 2,228,277 | 4,308,002 |
| Bayou Cocodrie National Wildlife Refuge | 9,989 | 134 | 65 | 6,626 | 30,181 | 438,225 | 1,460,750 | 2,191,125 | 4,236,176 |
| Necedah National Wildlife Refuge | 16,827 | 45 | 72 | 13,744 | 29,495 | 428,260 | 1,427,534 | 2,141,301 | 4,139,849 |
| Bond Swamp National Wildlife Refuge | 15,762 | 282 | 180 | 10,130 | 29,232 | 424,448 | 1,414,826 | 2,122,239 | 4,102,995 |
| Lacassine National Wildlife Refuge | 10,231 | 58 | 88 | 10,587 | 29,077 | 422,194 | 1,407,314 | 2,110,971 | 4,081,212 |
| San Luis National Wildlife Refuge | 4,550 | 109 | 108 | 4,509 | 28,787 | 417,993 | 1,393,310 | 2,089,965 | 4,040,600 |
| Cat Island National Wildlife Refuge | 8,474 | 179 | 152 | 11,540 | 28,566 | 414,784 | 1,382,612 | 2,073,918 | 4,009,575 |
| Grand Bay National Wildlife Refuge | 14,750 | 185 | 207 | 11,772 | 28,558 | 414,664 | 1,382,215 | 2,073,322 | 4,008,422 |
| Clear Lake National Wildlife Refuge | 2,493 | 78 | 74 | 3,671 | 28,090 | 407,861 | 1,359,538 | 2,039,307 | 3,942,661 |
| Cape May National Wildlife Refuge | 14,346 | 154 | 187 | 15,948 | 27,802 | 403,683 | 1,345,611 | 2,018,417 | 3,902,272 |
| J. N. Ding Darling National Wildlife Refuge | 7,062 | 283 | 77 | 8,014 | 27,449 | 398,556 | 1,328,520 | 1,992,779 | 3,852,707 |
| Rappahannock River Valley National Wildlife Refuge | 13,679 | 187 | 165 | 10,408 | 26,398 | 383,296 | 1,277,653 | 1,916,479 | 3,705,194 |
| Crocodile Lake National Wildlife Refuge | 24,847 | 263 | 177 | 16,697 | 26,292 | 381,762 | 1,272,539 | 1,908,809 | 3,690,364 |
| Ernest F. Hollings Ace Basin National Wildlife Refuge | 11,678 | 139 | 225 | 16,203 | 24,834 | 360,591 | 1,201,971 | 1,802,956 | 3,485,715 |
| Great River National Wildlife Refuge | 10,621 | 141 | 64 | 7,430 | 24,820 | 360,387 | 1,201,291 | 1,801,937 | 3,483,745 |
| Sutter National Wildlife Refuge | 10,380 | 626 | 498 | 9,871 | 24,709 | 358,781 | 1,195,937 | 1,793,906 | 3,468,218 |
| Breton National Wildlife Refuge | 4,825 | 55 | 55 | 4,825 | 23,013 | 334,146 | 1,113,820 | 1,670,730 | 3,230,077 |
| Cameron Prairie National Wildlife Refuge | 11,436 | 63 | 74 | 13,895 | 22,941 | 333,097 | 1,110,323 | 1,665,484 | 3,219,937 |
| Bald Knob National Wildlife Refuge | 4,490 | 100 | 109 | 4,442 | 22,854 | 331,847 | 1,106,156 | 1,659,235 | 3,207,854 |
| Bayou Teche National Wildlife Refuge | 13,242 | 172 | 141 | 9,979 | 22,823 | 331,389 | 1,104,631 | 1,656,947 | 3,203,431 |
| Caddo Lake National Wildlife Refuge | 9,978 | 198 | 99 | 8,338 | 22,748 | 330,300 | 1,101,000 | 1,651,500 | 3,192,900 |
| National Bison Range | 5,159 | 80 | 79 | 5,059 | 22,372 | 324,836 | 1,082,788 | 1,624,182 | 3,140,086 |
| San Joaquin River National Wildlife Refuge | 4,391 | 132 | 86 | 3,961 | 22,148 | 321,594 | 1,071,980 | 1,607,970 | 3,108,741 |
| Sequoyah National Wildlife Refuge | 4,575 | 71 | 59 | 3,963 | 22,129 | 321,311 | 1,071,038 | 1,606,557 | 3,106,011 |
| Lost Trail National Wildlife Refuge | 10,781 | 166 | 153 | 10,658 | 22,071 | 320,478 | 1,068,259 | 1,602,389 | 3,097,952 |
| Cypress Creek National Wildlife Refuge | 5,204 | 87 | 142 | 6,814 | 21,963 | 318,904 | 1,063,013 | 1,594,519 | 3,082,737 |
| Morgan Brake National Wildlife Refuge | 10,137 | 193 | 188 | 7,350 | 21,689 | 314,929 | 1,049,765 | 1,574,647 | 3,044,318 |
| Turnbull National Wildlife Refuge | 7,200 | 82 | 65 | 5,969 | 21,642 | 314,246 | 1,047,485 | 1,571,228 | 3,037,708 |
| Harris Neck National Wildlife Refuge | 37,770 | 512 | 214 | 17,436 | 21,435 | 311,240 | 1,037,467 | 1,556,200 | 3,008,654 |
| Two Rivers National Wildlife Refuge | 16,888 | 189 | 45 | 6,316 | 21,163 | 307,285 | 1,024,285 | 1,536,427 | 2,970,426 |
| Canaan Valley National Wildlife Refuge | 11,383 | 86 | 139 | 12,036 | 21,129 | 306,793 | 1,022,644 | 1,533,966 | 2,965,667 |
| Overflow National Wildlife Refuge | 6,628 | 104 | 118 | 7,399 | 21,045 | 305,573 | 1,018,576 | 1,527,865 | 2,953,871 |
| Bear Lake National Wildlife Refuge | 10,572 | 77 | 7 | 7,481 | 20,815 | 302,231 | 1,007,436 | 1,511,154 | 2,921,565 |
| Ruby Lake National Wildlife Refuge | 3,613 | 34 | 39 | 3,074 | 20,376 | 295,862 | 986,208 | 1,479,312 | 2,860,003 |
| Salt Plains National Wildlife Refuge | 1,939 | 42 | 55 | 3,365 | 19,884 | 288,716 | 962,388 | 1,443,582 | 2,790,924 |
| Rachel Carson National Wildlife Refuge | 16,058 | 227 | 217 | 15,078 | 19,274 | 279,856 | 932,855 | 1,399,282 | 2,705,279 |
| Great Swamp National Wildlife Refuge | 19,967 | 164 | 120 | 12,335 | 18,953 | 275,204 | 917,348 | 1,376,022 | 2,660,310 |
| Deep Fork National Wildlife Refuge | 7,935 | 114 | 138 | 6,064 | 17,460 | 253,515 | 845,052 | 1,267,577 | 2,450,650 |
| James River National Wildlife Refuge | 16,909 | 272 | 205 | 12,705 | 17,446 | 253,315 | 844,384 | 1,266,576 | 2,448,714 |
| Bear Valley National Wildlife Refuge | 10,647 | 270 | 235 | 11,751 | 16,891 | 245,256 | 817,520 | 1,226,281 | 2,370,809 |
| Muscatatuck National Wildlife Refuge | 9,067 | 142 | 72 | 7,427 | 16,487 | 239,397 | 797,989 | 1,196,984 | 2,314,169 |
| Patoka River National Wildlife Refuge | 7,722 | 109 | 106 | 6,948 | 16,470 | 239,140 | 797,133 | 1,195,700 | 2,311,687 |
| Dahomey National Wildlife Refuge | 7,822 | 108 | 168 | 8,685 | 15,734 | 228,457 | 761,523 | 1,142,285 | 2,208,418 |
| Bombay Hook National Wildlife Refuge | 13,422 | 69 | 56 | 10,043 | 15,656 | 227,332 | 757,772 | 1,136,659 | 2,197,540 |
| Patuxent Research Refuge | 13,602 | 82 | (102) | 6,627 | 15,628 | 226,926 | 756,419 | 1,134,629 | 2,193,615 |
| Desoto National Wildlife Refuge | 4,851 | 134 | 48 | 4,914 | 15,525 | 225,420 | 751,401 | 1,127,102 | 2,179,064 |
| Eufaula National Wildlife Refuge | 7,860 | 94 | 129 | 6,874 | 15,426 | 223,980 | 746,601 | 1,119,902 | 2,165,143 |
| Cahaba River National Wildlife Refuge | 11,568 | 284 | 245 | 9,760 | 15,240 | 221,288 | 737,626 | 1,106,439 | 2,139,115 |
| Oyster Bay National Wildlife Refuge | 11,960 | 303 | (93) | 6,067 | 15,171 | 220,287 | 734,290 | 1,101,435 | 2,129,442 |
| Bitter Creek National Wildlife Refuge | 3,511 | 67 | 70 | 3,643 | 14,459 | 209,948 | 699,827 | 1,049,740 | 2,029,498 |
| San Diego National Wildlife Refuge | 3,314 | 81 | 51 | 3,506 | 14,403 | 209,130 | 697,100 | 1,045,650 | 2,021,590 |
| Choctaw National Wildlife Refuge | 12,258 | 241 | 224 | 10,479 | 14,209 | 206,311 | 687,704 | 1,031,556 | 1,994,341 |
| Big Muddy National Fish And Wildlife Refuge | 6,715 | 53 | 61 | 5,344 | 14,199 | 206,166 | 687,219 | 1,030,828 | 1,992,935 |
| Wapanocca National Wildlife Refuge | 6,794 | 164 | 268 | 9,230 | 13,671 | 198,507 | 661,691 | 992,536 | 1,918,903 |
| Tamarac National Wildlife Refuge | 12,520 | 21 | 10 | 10,190 | 13,422 | 194,892 | 649,640 | 974,460 | 1,883,957 |
| St. Johns National Wildlife Refuge | 13,186 | 140 | 73 | 12,870 | 13,400 | 194,572 | 648,573 | 972,859 | 1,880,861 |
| Tule Lake National Wildlife Refuge | 9,016 | 23 | 30 | 5,409 | 13,004 | 188,822 | 629,407 | 944,111 | 1,825,281 |
| Erie National Wildlife Refuge | 9,761 | 99 | 71 | 10,564 | 12,953 | 188,071 | 626,904 | 940,356 | 1,818,022 |
| Wallkill River National Wildlife Refuge | 14,170 | 143 | 72 | 10,951 | 12,735 | 184,914 | 616,381 | 924,572 | 1,787,506 |
| Neches River National Wildlife Refuge | 8,008 | 127 | 113 | 7,876 | 12,735 | 184,911 | 616,371 | 924,557 | 1,787,477 |
| Stone Lakes National Wildlife Refuge | 6,354 | 134 | 70 | 3,639 | 12,692 | 184,294 | 614,313 | 921,470 | 1,781,508 |
| Middle Mississippi River National Wildlife Refuge | 9,643 | 106 | 97 | 8,279 | 12,687 | 184,209 | 614,029 | 921,043 | 1,780,683 |
| Aroostook National Wildlife Refuge | 11,795 | 162 | 163 | 14,300 | 12,644 | 183,594 | 611,981 | 917,972 | 1,774,746 |
| Green Bay National Wildlife Refuge | 8,215 | 507 | 282 | 7,563 | 12,461 | 180,928 | 603,092 | 904,639 | 1,748,968 |
| Chassahowitzka National Wildlife Refuge | 16,102 | 29 | 38 | 17,291 | 12,456 | 180,857 | 602,856 | 904,284 | 1,748,282 |
| Great Meadows National Wildlife Refuge | 12,759 | 216 | 146 | 13,172 | 12,403 | 180,086 | 600,288 | 900,432 | 1,740,835 |
| Umatilla National Wildlife Refuge | 2,372 | 35 | 43 | 2,388 | 12,312 | 178,765 | 595,883 | 893,825 | 1,728,061 |
| Conboy Lake National Wildlife Refuge | 8,196 | 127 | 183 | 10,395 | 12,224 | 177,500 | 591,665 | 887,498 | 1,715,830 |
| Little Sandy National Wildlife Refuge | 9,039 | 210 | 126 | 5,916 | 11,889 | 172,635 | 575,451 | 863,176 | 1,668,808 |
| Leslie Canyon National Wildlife Refuge | 2,015 | 34 | 16 | 2,083 | 11,773 | 170,944 | 569,815 | 854,722 | 1,652,463 |
| Arapaho National Wildlife Refuge | 3,369 | 31 | 68 | 4,129 | 11,718 | 170,148 | 567,161 | 850,741 | 1,644,766 |
| Wertheim National Wildlife Refuge | 13,136 | 296 | 21 | 4,824 | 11,691 | 169,758 | 565,861 | 848,792 | 1,640,998 |
| Northern Tallgrass Prairie National Wildlife Refuge | 7,492 | 77 | 25 | 8,637 | 11,281 | 163,800 | 545,999 | 818,999 | 1,583,398 |
| Pixley National Wildlife Refuge | 2,915 | 100 | 84 | 2,684 | 11,010 | 159,872 | 532,907 | 799,361 | 1,545,431 |
| Red River National Wildlife Refuge | 4,910 | 55 | 57 | 5,449 | 10,903 | 158,314 | 527,712 | 791,568 | 1,530,366 |
| Yazoo National Wildlife Refuge | 2,711 | 56 | 60 | 4,961 | 10,870 | 157,839 | 526,130 | 789,196 | 1,525,778 |
| Columbia National Wildlife Refuge | 2,800 | 25 | 35 | 2,689 | 10,835 | 157,319 | 524,398 | 786,597 | 1,520,755 |
| Nine-Pipe National Wildlife Refuge | 4,438 | 349 | 99 | 5,690 | 10,796 | 156,751 | 522,504 | 783,756 | 1,515,261 |
| Black Bayou Lake National Wildlife Refuge | 9,132 | 135 | 105 | 9,197 | 10,569 | 153,463 | 511,545 | 767,317 | 1,483,480 |
| Shiawassee National Wildlife Refuge | 7,163 | 67 | 37 | 8,406 | 10,047 | 145,885 | 486,285 | 729,427 | 1,410,226 |
| Billy Frank Jr. Nisqually National Wildlife Refuge | 13,662 | 133 | 116 | 11,569 | 9,836 | 142,822 | 476,074 | 714,110 | 1,380,613 |
| Ozark Plateau National Wildlife Refuge | 7,290 | 155 | 129 | 6,289 | 9,783 | 142,051 | 473,502 | 710,253 | 1,373,157 |
| Grays Harbor National Wildlife Refuge | 24,796 | 466 | 120 | 15,944 | 9,771 | 141,876 | 472,920 | 709,379 | 1,371,467 |
| Swanquarter National Wildlife Refuge | 20,848 | 39 | 38 | 16,412 | 9,601 | 139,407 | 464,691 | 697,037 | 1,347,604 |
| Mcnary National Wildlife Refuge | 2,299 | 40 | 46 | 2,583 | 9,382 | 136,223 | 454,077 | 681,115 | 1,316,822 |
| Modoc National Wildlife Refuge | 3,766 | 86 | 49 | 4,071 | 9,087 | 131,943 | 439,809 | 659,714 | 1,275,447 |
| Banks Lake National Wildlife Refuge | 15,907 | 204 | 203 | 10,162 | 9,017 | 130,924 | 436,412 | 654,618 | 1,265,595 |
| Lacreek National Wildlife Refuge | 3,152 | 36 | 23 | 2,976 | 8,876 | 128,880 | 429,599 | 644,399 | 1,245,838 |
| Fort Niobrara National Wildlife Refuge | 2,301 | 31 | 29 | 2,628 | 8,763 | 127,239 | 424,131 | 636,197 | 1,229,981 |
| Buenos Aires National Wildlife Refuge | 2,906 | 5 | 11 | 2,645 | 8,547 | 124,106 | 413,687 | 620,531 | 1,199,692 |
| Minidoka National Wildlife Refuge | 2,286 | 26 | 26 | 2,606 | 8,522 | 123,736 | 412,453 | 618,679 | 1,196,113 |
| Deer Flat National Wildlife Refuge | 3,803 | 49 | 28 | 3,303 | 8,505 | 123,489 | 411,630 | 617,445 | 1,193,727 |
| Bitter Lake National Wildlife Refuge | 2,278 | 22 | 23 | 2,294 | 8,226 | 119,447 | 398,155 | 597,233 | 1,154,650 |
| Merced National Wildlife Refuge | 3,402 | 143 | 95 | 3,544 | 8,103 | 117,659 | 392,197 | 588,295 | 1,137,370 |
| Big Boggy National Wildlife Refuge | 8,461 | 120 | 124 | 7,161 | 8,039 | 116,722 | 389,072 | 583,608 | 1,128,309 |
| Ridgefield National Wildlife Refuge | 11,881 | 107 | 59 | 11,081 | 8,009 | 116,293 | 387,644 | 581,467 | 1,124,169 |
| Don Edwards San Francisco Bay National Wildlife Refuge | 2,771 | 18 | 6 | 3,827 | 7,928 | 115,121 | 383,735 | 575,603 | 1,112,832 |
| Havasu National Wildlife Refuge | 958 | 13 | 18 | 1,145 | 7,768 | 112,784 | 375,948 | 563,921 | 1,090,248 |
| Kern National Wildlife Refuge | 2,261 | 46 | 46 | 2,382 | 7,677 | 111,467 | 371,555 | 557,333 | 1,077,510 |
| Moody National Wildlife Refuge | 9,449 | 147 | 110 | 7,501 | 7,661 | 111,239 | 370,796 | 556,193 | 1,075,307 |
| Quivira National Wildlife Refuge | 3,343 | 23 | 38 | 2,621 | 7,635 | 110,853 | 369,511 | 554,267 | 1,071,582 |
| Wapack National Wildlife Refuge | 14,722 | 302 | 232 | 13,557 | 7,363 | 106,910 | 356,366 | 534,549 | 1,033,461 |
| Las Vegas National Wildlife Refuge | 3,332 | 56 | 37 | 3,341 | 7,251 | 105,277 | 350,924 | 526,386 | 1,017,680 |
| Ohio River Islands National Wildlife Refuge | 8,038 | 147 | 136 | 9,057 | 7,219 | 104,819 | 349,398 | 524,097 | 1,013,254 |
| Alamosa National Wildlife Refuge | 5,108 | 40 | 46 | 4,260 | 7,196 | 104,480 | 348,268 | 522,402 | 1,009,976 |
| Cibola National Wildlife Refuge | 1,677 | 26 | 86 | 1,772 | 7,157 | 103,917 | 346,389 | 519,583 | 1,004,527 |
| Waubay National Wildlife Refuge | 6,140 | 104 | 59 | 7,646 | 7,149 | 103,805 | 346,016 | 519,024 | 1,003,447 |
| Lake Wales Ridge National Wildlife Refuge | 16,890 | 248 | 94 | 11,749 | 6,977 | 101,309 | 337,698 | 506,547 | 979,324 |
| Boyer Chute National Wildlife Refuge | 5,078 | 118 | 65 | 5,666 | 6,942 | 100,798 | 335,994 | 503,991 | 974,383 |
| Cedar Island National Wildlife Refuge | 16,573 | 32 | 53 | 17,049 | 6,824 | 99,083 | 330,278 | 495,417 | 957,807 |
| Bosque Del Apache National Wildlife Refuge | 1,844 | 8 | 9 | 1,911 | 6,725 | 97,652 | 325,507 | 488,261 | 943,971 |
| Arrowwood National Wildlife Refuge | 6,490 | 33 | (6) | 7,190 | 6,603 | 95,872 | 319,573 | 479,359 | 926,761 |
| Tallahatchie National Wildlife Refuge | 7,581 | 106 | 85 | 5,760 | 6,584 | 95,604 | 318,680 | 478,020 | 924,172 |
| Tetlin National Wildlife Refuge | 26,805 | 1 | 6 | 24,231 | 6,451 | 93,673 | 312,242 | 468,363 | 905,502 |
| Attwater Prairie Chicken National Wildlife Refuge | 2,605 | 39 | 42 | 2,759 | 6,064 | 88,052 | 293,508 | 440,262 | 851,172 |
| Port Louisa National Wildlife Refuge | 7,174 | 22 | 84 | 8,447 | 5,712 | 82,934 | 276,446 | 414,669 | 801,693 |
| Texas Point National Wildlife Refuge | 7,951 | 46 | 45 | 16,137 | 5,629 | 81,729 | 272,429 | 408,644 | 790,045 |
| Holla Bend National Wildlife Refuge | 2,402 | 62 | 68 | 4,260 | 5,563 | 80,781 | 269,269 | 403,903 | 780,880 |
| Grand Cote National Wildlife Refuge | 6,804 | 63 | 97 | 6,394 | 5,531 | 80,311 | 267,703 | 401,554 | 776,338 |
| Wolf Island National Wildlife Refuge | 24,446 | 77 | 141 | 21,025 | 5,301 | 76,963 | 256,544 | 384,817 | 743,979 |
| Kirwin National Wildlife Refuge | 4,391 | 33 | 46 | 3,823 | 5,292 | 76,833 | 256,109 | 384,164 | 742,717 |
| Flint Hills National Wildlife Refuge | 6,316 | 19 | (8) | 6,484 | 5,183 | 75,261 | 250,868 | 376,303 | 727,519 |
| Chautauqua National Wildlife Refuge | 8,107 | 51 | 30 | 6,399 | 5,049 | 73,306 | 244,352 | 366,528 | 708,621 |
| Wassaw National Wildlife Refuge | 20,548 | 31 | 77 | 18,071 | 4,700 | 68,250 | 227,498 | 341,248 | 659,745 |
| Hanalei National Wildlife Refuge | 39 | 344 | 0 | 27 | 4,694 | 68,163 | 227,209 | 340,813 | 658,906 |
| Rocky Mountain Arsenal National Wildlife Refuge | 3,164 | 20 | (27) | 2,606 | 4,670 | 67,801 | 226,005 | 339,007 | 655,413 |
| Big Stone National Wildlife Refuge | 9,264 | 27 | 14 | 8,255 | 4,647 | 67,468 | 224,893 | 337,340 | 652,191 |
| Back Bay National Wildlife Refuge | 9,956 | 36 | 128 | 8,889 | 4,623 | 67,121 | 223,735 | 335,603 | 648,832 |
| Mason Neck National Wildlife Refuge | 12,758 | 135 | 59 | 12,045 | 4,570 | 66,359 | 221,196 | 331,794 | 641,469 |
| Loess Bluffs National Wildlife Refuge | 7,142 | 40 | 25 | 5,999 | 4,491 | 65,208 | 217,360 | 326,041 | 630,345 |
| Assabet River National Wildlife Refuge | 16,711 | 126 | 161 | 15,075 | 4,400 | 63,886 | 212,955 | 319,432 | 617,569 |
| Montezuma National Wildlife Refuge | 16,150 | 29 | 34 | 12,159 | 4,362 | 63,343 | 211,142 | 316,713 | 612,312 |
| Buffalo Lake National Wildlife Refuge | 2,186 | 37 | 20 | 2,650 | 4,207 | 61,092 | 203,641 | 305,462 | 590,559 |
| Theodore Roosevelt National Wildlife Refuge | 3,943 | 45 | 56 | 4,771 | 4,173 | 60,596 | 201,986 | 302,979 | 585,760 |
| Maxwell National Wildlife Refuge | 2,428 | 78 | (2) | 2,358 | 4,152 | 60,286 | 200,953 | 301,430 | 582,765 |
| James Campbell National Wildlife Refuge | 41 | 259 | 0 | 26 | 4,112 | 59,713 | 199,043 | 298,564 | 577,224 |
| Neal Smith National Wildlife Refuge | 5,326 | 49 | 64 | 6,358 | 4,094 | 59,438 | 198,126 | 297,189 | 574,566 |
| Nestucca Bay National Wildlife Refuge | 40,366 | 222 | 241 | 30,591 | 3,959 | 57,482 | 191,608 | 287,412 | 555,663 |
| Ottawa National Wildlife Refuge | 9,954 | 37 | 20 | 7,000 | 3,901 | 56,641 | 188,805 | 283,207 | 547,533 |
| Holt Collier National Wildlife Refuge | 9,846 | 170 | 52 | 3,728 | 3,894 | 56,547 | 188,489 | 282,733 | 546,617 |
| Baca National Wildlife Refuge | 2,441 | 3 | 11 | 2,692 | 3,647 | 52,949 | 176,497 | 264,745 | 511,841 |
| Cherry Valley National Wildlife Refuge | 15,513 | 111 | 105 | 11,978 | 3,553 | 51,589 | 171,963 | 257,945 | 498,694 |
| Union Slough National Wildlife Refuge | 9,447 | 81 | 38 | 10,525 | 3,495 | 50,746 | 169,153 | 253,729 | 490,542 |
| Rice Lake National Wildlife Refuge | 12,072 | 13 | 49 | 14,298 | 3,467 | 50,340 | 167,801 | 251,702 | 486,623 |
| Washita National Wildlife Refuge | 2,973 | 29 | 23 | 2,619 | 3,451 | 50,109 | 167,029 | 250,544 | 484,385 |
| Detroit River International Wildlife Refuge | 5,197 | 42 | (13) | 7,064 | 3,427 | 49,754 | 165,846 | 248,770 | 480,955 |
| Iroquois National Wildlife Refuge | 13,286 | 21 | 14 | 10,483 | 3,391 | 49,231 | 164,105 | 246,157 | 475,904 |
| Optima National Wildlife Refuge | 3,406 | 52 | 55 | 3,020 | 3,358 | 48,759 | 162,528 | 243,793 | 471,333 |
| Mandalay National Wildlife Refuge | 9,000 | 49 | 34 | 7,685 | 3,341 | 48,508 | 161,693 | 242,539 | 468,909 |
| Ash Meadows National Wildlife Refuge | 1,107 | 9 | 14 | 1,179 | 3,231 | 46,913 | 156,378 | 234,567 | 453,496 |
| Seedskadee National Wildlife Refuge | 1,684 | 8 | 10 | 1,674 | 3,152 | 45,773 | 152,577 | 228,865 | 442,473 |
| Fallon National Wildlife Refuge | 1,254 | 12 | 7 | 1,154 | 3,147 | 45,697 | 152,323 | 228,485 | 441,737 |
| Cold Springs National Wildlife Refuge | 2,597 | 65 | 11 | 2,457 | 3,000 | 43,560 | 145,200 | 217,800 | 421,080 |
| Muleshoe National Wildlife Refuge | 2,484 | 28 | 7 | 2,341 | 2,924 | 42,462 | 141,539 | 212,309 | 410,463 |
| Oxbow National Wildlife Refuge | 25,867 | 115 | 157 | 14,761 | 2,912 | 42,276 | 140,921 | 211,381 | 408,670 |
| Ouray National Wildlife Refuge | 1,783 | 21 | 1 | 1,618 | 2,904 | 42,171 | 140,570 | 210,855 | 407,654 |
| Coachella Valley National Wildlife Refuge | 1,638 | 48 | 8 | 1,819 | 2,606 | 37,833 | 126,110 | 189,164 | 365,718 |
| Supawna Meadows National Wildlife Refuge | 17,917 | 50 | 50 | 11,716 | 2,466 | 35,801 | 119,338 | 179,007 | 346,081 |
| Minnesota Valley National Wildlife Refuge | 8,456 | 11 | (29) | 7,267 | 2,426 | 35,219 | 117,396 | 176,094 | 340,449 |
| William L. Finley National Wildlife Refuge | 7,654 | 28 | 8 | 7,225 | 2,355 | 34,197 | 113,988 | 170,983 | 330,567 |
| Bowdoin National Wildlife Refuge | 3,315 | 10 | 9 | 2,597 | 2,352 | 34,149 | 113,831 | 170,747 | 330,110 |
| Meredosia National Wildlife Refuge | 8,068 | 43 | 32 | 5,857 | 2,335 | 33,901 | 113,002 | 169,503 | 327,707 |
| Sherburne National Wildlife Refuge | 13,972 | 5 | 57 | 10,220 | 2,303 | 33,435 | 111,448 | 167,173 | 323,200 |
| Long Lake National Wildlife Refuge | 5,876 | 11 | 6 | 5,251 | 2,272 | 32,985 | 109,950 | 164,925 | 318,856 |
| Bill Williams River National Wildlife Refuge | 893 | 24 | 39 | 1,135 | 2,135 | 30,994 | 103,313 | 154,969 | 299,607 |
| Benton Lake National Wildlife Refuge | 2,484 | 12 | 3 | 2,471 | 2,113 | 30,675 | 102,251 | 153,377 | 296,529 |
| Cedar Point National Wildlife Refuge | 9,888 | 49 | 18 | 6,854 | 1,891 | 27,453 | 91,512 | 137,267 | 265,384 |
| Slade National Wildlife Refuge | 5,607 | 42 | 14 | 4,284 | 1,852 | 26,888 | 89,628 | 134,442 | 259,921 |
| Sullys Hill National Game Preserve | 6,008 | 70 | 16 | 7,726 | 1,680 | 24,394 | 81,314 | 121,970 | 235,809 |
| Crane Meadows National Wildlife Refuge | 8,951 | 56 | 17 | 10,208 | 1,631 | 23,685 | 78,951 | 118,427 | 228,959 |
| Stewart B. Mckinney National Wildlife Refuge | 8,133 | 105 | 109 | 14,547 | 1,622 | 23,549 | 78,497 | 117,745 | 227,641 |
| Pathfinder National Wildlife Refuge | 2,816 | 6 | 16 | 2,436 | 1,416 | 20,566 | 68,552 | 102,828 | 198,800 |
| Missisquoi National Wildlife Refuge | 8,168 | 12 | 38 | 12,472 | 1,339 | 19,436 | 64,785 | 97,178 | 187,878 |
| North Platte National Wildlife Refuge | 2,296 | 30 | 27 | 2,776 | 1,313 | 19,064 | 63,546 | 95,319 | 184,283 |
| Grays Lake National Wildlife Refuge | 6,328 | 20 | 50 | 7,415 | 1,304 | 18,933 | 63,110 | 94,666 | 183,020 |
| Camas National Wildlife Refuge | 3,355 | 8 | 34 | 2,319 | 1,289 | 18,716 | 62,387 | 93,580 | 180,922 |
| Coldwater River National Wildlife Refuge | 3,306 | 31 | 62 | 4,289 | 1,154 | 16,754 | 55,848 | 83,772 | 161,959 |
| Key Cave National Wildlife Refuge | 2,050 | 69 | 93 | 5,179 | 1,072 | 15,566 | 51,888 | 77,832 | 150,475 |
| Mortenson Lake National Wildlife Refuge | 1,931 | 36 | 32 | 2,443 | 1,038 | 15,071 | 50,237 | 75,355 | 145,686 |
| Hutton Lake National Wildlife Refuge | 2,361 | 35 | 31 | 2,475 | 1,030 | 14,961 | 49,870 | 74,805 | 144,623 |
| Prime Hook National Wildlife Refuge | 3,130 | 7 | 82 | 13,335 | 1,030 | 14,960 | 49,867 | 74,800 | 144,613 |
| Pahranagat National Wildlife Refuge | 1,559 | 14 | 19 | 1,482 | 935 | 13,573 | 45,244 | 67,866 | 131,208 |
| Grulla National Wildlife Refuge | 2,178 | 18 | 24 | 2,274 | 887 | 12,883 | 42,945 | 64,417 | 124,540 |
| Cokeville Meadows National Wildlife Refuge | 5,456 | 8 | 1 | 4,327 | 790 | 11,467 | 38,224 | 57,336 | 110,850 |
| John W. And Louise Seier National Wildlife Refuge | 2,957 | 22 | 23 | 2,783 | 777 | 11,278 | 37,593 | 56,389 | 109,020 |
| Presquile National Wildlife Refuge | 10,268 | 38 | (46) | 7,430 | 731 | 10,611 | 35,369 | 53,054 | 102,570 |
| Pinckney Island National Wildlife Refuge | 2,475 | 12 | 135 | 10,515 | 709 | 10,291 | 34,302 | 51,453 | 99,477 |
| Imperial National Wildlife Refuge | 1,656 | 1 | 3 | 1,051 | 630 | 9,154 | 30,515 | 45,772 | 88,492 |
| Dungeness National Wildlife Refuge | 9,299 | 29 | 81 | 10,213 | 609 | 8,843 | 29,476 | 44,213 | 85,479 |
| Lake Isom National Wildlife Refuge | 3,199 | 22 | 267 | 10,785 | 595 | 8,638 | 28,794 | 43,191 | 83,503 |
| War Horse National Wildlife Refuge | 2,680 | 14 | 8 | 2,657 | 591 | 8,575 | 28,583 | 42,875 | 82,892 |
| Steigerwald Lake National Wildlife Refuge | 8,072 | 37 | 101 | 16,030 | 580 | 8,427 | 28,089 | 42,134 | 81,459 |
| Rydell National Wildlife Refuge | 10,529 | 19 | (4) | 8,092 | 561 | 8,143 | 27,142 | 40,713 | 78,712 |
| Driftless Area National Wildlife Refuge | 5,611 | 25 | 51 | 6,966 | 444 | 6,446 | 21,486 | 32,228 | 62,308 |
| Tualatin River National Wildlife Refuge | 6,878 | 18 | 26 | 8,901 | 353 | 5,127 | 17,092 | 25,637 | 49,565 |
| Chase Lake National Wildlife Refuge | 5,011 | 4 | (6) | 6,396 | 260 | 3,775 | 12,584 | 18,876 | 36,493 |
| Hakalau Forest National Wildlife Refuge | 49 | 0 | 0 | 45 | 246 | 3,578 | 11,928 | 17,891 | 34,590 |
| Santa Ana National Wildlife Refuge | 2,975 | 7 | 68 | 2,801 | 224 | 3,247 | 10,822 | 16,234 | 31,385 |
| Grass Lake National Wildlife Refuge | 2,777 | 4 | 2 | 2,930 | 211 | 3,059 | 10,195 | 15,293 | 29,566 |
| White Lake National Wildlife Refuge | 3,514 | 2 | (4) | 3,641 | 34 | 488 | 1,627 | 2,441 | 4,719 |
| Baskett Slough National Wildlife Refuge | 5,525 | 1 | 43 | 8,224 | 33 | 473 | 1,576 | 2,364 | 4,570 |
| Oahu Forest National Wildlife Refuge | 56 | 0 | 0 | 44 | 25 | 368 | 1,227 | 1,841 | 3,559 |
| Monte Vista National Wildlife Refuge | 3,346 | 0 | 33 | 2,828 | 17 | 247 | 825 | 1,237 | 2,392 |
| Lee Metcalf National Wildlife Refuge | 4,469 | 0 | (31) | 4,673 | 8 | 111 | 370 | 555 | 1,074 |
| Emiquon National Wildlife Refuge | 6,080 | (0) | 28 | 6,308 | -5 | -78 | -260 | -390 | -754 |
| Clarence Cannon National Wildlife Refuge | 4,871 | (1) | 34 | 5,932 | -62 | -905 | -3,017 | -4,525 | -8,748 |
| Ankeny National Wildlife Refuge | 5,150 | (2) | 10 | 7,893 | -82 | -1,197 | -3,991 | -5,987 | -11,575 |
| Lake Andes National Wildlife Refuge | 6,112 | (10) | 48 | 5,503 | -161 | -2,341 | -7,803 | -11,704 | -22,628 |
| Lake Ilo National Wildlife Refuge | 3,727 | (3) | (11) | 4,155 | -187 | -2,712 | -9,039 | -13,558 | -26,212 |
| Tewaukon National Wildlife Refuge | 9,663 | (3) | 1 | 8,828 | -309 | -4,480 | -14,932 | -22,398 | -43,304 |
| Lake Zahl National Wildlife Refuge | 4,367 | (7) | (8) | 4,662 | -327 | -4,742 | -15,806 | -23,708 | -45,836 |
| Florence Lake National Wildlife Refuge | 5,860 | (16) | (18) | 5,628 | -351 | -5,091 | -16,968 | -25,453 | -49,208 |
| John Heinz National Wildlife Refuge At Tinicum | 2,123 | (28) | 51 | 5,313 | -424 | -6,153 | -20,508 | -30,763 | -59,475 |
| Wapato Lake National Wildlife Refuge | 18,502 | (36) | 39 | 9,708 | -493 | -7,153 | -23,844 | -35,767 | -69,149 |
| Karl E. Mundt National Wildlife Refuge | 4,405 | (32) | 23 | 4,456 | -517 | -7,510 | -25,035 | -37,552 | -72,600 |
| Fox River National Wildlife Refuge | 14,868 | (56) | 12 | 11,279 | -831 | -12,071 | -40,236 | -60,354 | -116,685 |
| Kootenai National Wildlife Refuge | 6,417 | (24) | 90 | 11,654 | -1,001 | -14,541 | -48,469 | -72,703 | -140,560 |
| Lake Nettie National Wildlife Refuge | 4,795 | (30) | (45) | 5,723 | -1,073 | -15,574 | -51,913 | -77,870 | -150,548 |
| Trempealeau National Wildlife Refuge | 2,916 | (13) | 37 | 8,002 | -1,272 | -18,476 | -61,587 | -92,380 | -178,601 |
| Rocky Flats National Wildlife Refuge | 3,264 | (16) | 11 | 3,719 | -1,275 | -18,517 | -61,725 | -92,587 | -179,001 |
| Lake Mason National Wildlife Refuge | 2,685 | (8) | 4 | 2,548 | -1,419 | -20,600 | -68,668 | -103,002 | -199,138 |
| Browns Park National Wildlife Refuge | 2,428 | (8) | 28 | 4,178 | -1,432 | -20,789 | -69,297 | -103,946 | -200,962 |
| Hamden Slough National Wildlife Refuge | 7,285 | (41) | (26) | 8,959 | -2,099 | -30,484 | -101,612 | -152,418 | -294,675 |
| Sabine National Wildlife Refuge | 12,846 | (1) | 35 | 13,384 | -2,220 | -32,240 | -107,468 | -161,202 | -311,658 |
| Fish Springs National Wildlife Refuge | 776 | (9) | (9) | 673 | -2,280 | -33,103 | -110,342 | -165,513 | -319,992 |
| Parker River National Wildlife Refuge | 14,485 | (38) | 90 | 15,264 | -2,564 | -37,224 | -124,081 | -186,122 | -359,835 |
| Lostwood National Wildlife Refuge | 5,143 | (7) | (9) | 5,295 | -2,644 | -38,395 | -127,985 | -191,977 | -371,155 |
| Glacial Ridge National Wildlife Refuge | 6,312 | (10) | (25) | 6,867 | -3,479 | -50,508 | -168,361 | -252,542 | -488,248 |
| Marais Des Cygnes National Wildlife Refuge | 15,174 | (33) | 79 | 6,900 | -3,689 | -53,566 | -178,553 | -267,829 | -517,804 |
| Valentine National Wildlife Refuge | 3,605 | (4) | 27 | 3,031 | -4,087 | -59,340 | -197,801 | -296,702 | -573,624 |
| Currituck National Wildlife Refuge | 19,700 | (33) | 29 | 16,102 | -4,262 | -61,891 | -206,303 | -309,454 | -598,278 |
| Lake Alice National Wildlife Refuge | 6,456 | (37) | (39) | 7,622 | -4,731 | -68,694 | -228,981 | -343,471 | -664,045 |
| Mackay Island National Wildlife Refuge | 14,775 | (43) | 135 | 13,313 | -5,537 | -80,399 | -267,996 | -401,994 | -777,188 |
| Medicine Lake National Wildlife Refuge | 3,835 | (13) | (14) | 4,158 | -6,008 | -87,239 | -290,797 | -436,195 | -843,310 |
| Red Rock Lakes National Wildlife Refuge | 4,797 | (7) | 27 | 5,879 | -6,631 | -96,277 | -320,923 | -481,385 | -930,678 |
| Des Lacs National Wildlife Refuge | 5,124 | (33) | (38) | 6,244 | -7,126 | -103,466 | -344,888 | -517,332 | -1,000,175 |
| Ul Bend National Wildlife Refuge | 2,840 | (9) | (13) | 2,803 | -7,389 | -107,292 | -357,641 | -536,462 | -1,037,159 |
| Audubon National Wildlife Refuge | 6,670 | (36) | (35) | 5,900 | -7,808 | -113,365 | -377,884 | -566,825 | -1,095,862 |
| Sand Lake National Wildlife Refuge | 6,373 | (32) | 20 | 7,337 | -9,429 | -136,911 | -456,369 | -684,554 | -1,323,471 |
| Mathews Brake National Wildlife Refuge | 9,450 | (269) | 79 | 6,251 | -9,541 | -138,530 | -461,768 | -692,652 | -1,339,127 |
| Horicon National Wildlife Refuge | 20,664 | (36) | (16) | 12,195 | -11,728 | -170,291 | -567,636 | -851,454 | -1,646,144 |
| Everglades Headwaters National Wildlife Refuge And Conservation Area | 6,933 | (144) | 95 | 10,782 | -13,469 | -195,577 | -651,923 | -977,884 | -1,890,576 |
| Big Oaks National Wildlife Refuge | 9,950 | (25) | 105 | 7,114 | -18,425 | -267,527 | -891,756 | -1,337,634 | -2,586,093 |
| Upper Souris National Wildlife Refuge | 6,289 | (42) | (49) | 7,097 | -19,915 | -289,173 | -963,910 | -1,445,865 | -2,795,339 |
| Bear River Migratory Bird Refuge | 2,142 | (20) | 12 | 3,899 | -23,190 | -336,718 | -1,122,392 | -1,683,589 | -3,254,938 |
| Hopper Mountain National Wildlife Refuge | 15,231 | (666) | (41) | 5,039 | -23,354 | -339,094 | -1,130,313 | -1,695,469 | -3,277,906 |
| Agassiz National Wildlife Refuge | 19,211 | (47) | (54) | 13,993 | -43,332 | -629,177 | -2,097,256 | -3,145,884 | -6,082,043 |
| J. Clark Salyer National Wildlife Refuge | 6,497 | (57) | (44) | 7,080 | -49,799 | -723,089 | -2,410,296 | -3,615,443 | -6,989,857 |
| Cabeza Prieta National Wildlife Refuge | 1,003 | (6) | (10) | 964 | -78,851 | -1,144,916 | -3,816,387 | -5,724,581 | -11,067,523 |
| Arthur R. Marshall Loxahatchee National Wildlife Refuge | 23,844 | (50) | (25) | 24,716 | -108,191 | -1,570,927 | -5,236,424 | -7,854,636 | -15,185,630 |
| Innoko National Wildlife Refuge | 19,502 | (4) | (3) | 21,653 | -223,456 | -3,244,580 | -10,815,266 | -16,222,898 | -31,364,270 |
| Charles M. Russell National Wildlife Refuge | 3,157 | (24) | (2) | 2,780 | -325,700 | -4,729,167 | -15,763,891 | -23,645,837 | -45,715,284 |
| Selawik National Wildlife Refuge | 39,106 | (17) | (9) | 35,801 | -665,113 | -9,657,447 | -32,191,490 | -48,287,234 | -93,355,320 |
| Koyukuk National Wildlife Refuge | 17,079 | (13) | (12) | 18,818 | -770,944 | -11,194,108 | -37,313,693 | -55,970,540 | -108,209,710 |
| Kanuti National Wildlife Refuge | 18,847 | (92) | (72) | 19,579 | -1,793,405 | -26,040,240 | -86,800,799 | -130,201,199 | -251,722,318 |
| Yukon Flats National Wildlife Refuge | 17,755 | (20) | 2 | 19,053 | -2,686,538 | -39,008,531 | -130,028,438 | -195,042,656 | -377,082,469 |
