# Supplementary material for: Conservation of carbon resources and values on public lands: A case study from the National Wildlife Refuge System
Source: PLoS One. 2022 Jan 12;17(1):e0262218. doi: 10.1371/journal.pone.0262218 (PMC8754292; doi:10.1371/journal.pone.0262218)
Supplement: Attachment — Submitted filename: RR_PONE-D-21-13289.doc [file pone.0262218.s002.doc]

**Review Report**

**Journal:** PLOS ONE

**Manuscript Number:** PONE-D-21-13289

**Title:** Conservation of carbon resources and values on public lands: A case study from the National Wildlife Refuge System

**Article Type:** Research paper

The paper is addressing an important aspect for climate change actions. The analysis may be relevant, however before publications the paper needs to be modified extensively. The inputs are attached below:

1. The language needs to be modified.
2. The editing for MS is desired. I suggest to see the MS for meticulous way. (Comma, mixing or joining or words etc.)
3. The abstract should explain the method.
4. The keywords should not include the words of the Title of MS.
5. Please define public lands focusing to the study.
6. Please include one para at the end of Introduction about the uses of the study.
7. The study area along with the detailed NWRS should be included as supplementary material. This may include longitude and latitude, major species, areas, year of establishment, management regime. etc.
8. Please provide details of two factors for ANOVA along with main effect and interaction.
9. Have you tested the assumptions of ANOVA specially linearity and homogeneity. Please elaborate.
10. Please elaborate the changes per unit area. Please provide the details of ANOVA.
11. Please see with longitude and latitude the changes are significant.
12. The discounting approach is straight however forests are dynamic. Please see if you can estimate the deforestation and degradation aspect of the forest. This can be achieved by analyzing the forest cover in successive years. This will add the dynamic components of the change.
13. How the social cost for future has been estimated. Business usual scenario may not exist in future. Please see if population changes or management regime changes. (what about fire as mentioned in the MS)
14. Can you discuss about the management of these lands, as the management may also influence the carbon capturing.
15. The ecosystem services (additional) may be discussed in terms of added economic gains, if possible.
16. Please add a section for conclusion.
17. Please add review of literature on the aspect in Introduction.
